# Bidirectional regulatory potentials of short-chain fatty acids and their G-protein-coupled receptors in autoimmune neuroinflammation

**DOI:** 10.1038/s41598-019-45311-y

**Published:** 2019-06-20

**Authors:** Jeongho Park, Qin Wang, Qi Wu, Yang Mao-Draayer, Chang H. Kim

**Affiliations:** 1 Department of Comparative Pathobiology, Purdue University, West Lafayette, IN 47907 USA; 2 Autoimmunity Center of Excellence, Multiple Sclerosis Center, Department of Neurology, University of Michigan Medical School, Ann Arbor, MI 48109 USA; 3 Laboratory of Immunology and Hematopoiesis, Department of Pathology and Mary H Weiser Food Allergy Center, University of Michigan School of Medicine, Ann Arbor, MI 48109 USA

**Keywords:** Inflammation, Chronic inflammation

## Abstract

Microbial metabolites, produced in the intestine, have significant effects on inflammatory diseases throughout the body. Short-chain fatty acids (SCFAs) have protective effects on experimental autoimmune encephalitis (EAE) responses but the detailed roles of SCFAs and their receptors in regulating autoimmune CNS inflammation have been unclear. SCFAs metabolically regulate T cells and change the phenotype of antigen presenting cells to efficiently induce IL-10^+^ regulatory T cells. In line with the overall protective effect, blood levels of major SCFAs, such as acetate, propionate and butyrate, are significantly decreased in long-term active progressive multiple sclerosis (MS) patients. Importantly, SCFAs can induce CD4^+^ effector T cells, which are highly inflammatory when transferred into mice, suggesting that the direct effect of SCFAs on T cells can even be pro-inflammatory in the CNS. In contrast to the moderate protective effect of SCFAs, mice deficient in GPR41 or GPR43 are more resistant to EAE pathogenesis. Thus, despite the overall protective function of SCFAs, SCFAs and their receptors have the potential to regulate autoimmune CNS inflammation both positively and negatively.

## Introduction

The mammalian intestine hosts hundreds of trillions of microbes, which produce a myriad of different metabolites^1,2^. The functions of these metabolites in regulating the host physiology and immune system have gained a lot of attention^3–12^. Short chain fatty acids (SCFAs) are mainly produced by bacterial fermentation of dietary fiber (DF) or glycosylated host proteins such as mucins in the colon. In addition, acetate (C2) is produced by host cells in the liver and other tissues^13^. SCFAs have many functions in regulating the gut immune system. SCFAs function through several different mechanisms, including metabolic integration, microbiota regulation, histone deacetylase (HDAC) inhibition, and G-protein coupled receptors (GPCRs) activation. SCFA activate multiple GPCRs such as GPR43, GPR41, GPR109A and Olfr78^14–17^, which are not expressed by lymphocytes at significant levels but are distinctively expressed by other cell types such as epithelial cells, myeloid cells and endothelial cells.

Multiple sclerosis (MS) is the most common autoimmune neuroinflammatory disease of the central nervous system (CNS), characterized by the infiltration of inflammatory cells, and demyelination and degeneration of brain and/or spinal cord tissues^18^. Major types of MS include clinically isolated syndrome (CIS), relapsing-remitting MS (RRMS), and secondary progressive MS (SPMS). SPMS patients develop gradual neurological deterioration with increasing physical debilitation superimposed on prolonged relapsing remitting conditions. Some patients develop primary-progressive MS (PPMS), which is defined as steady disease progression from its onset without the relapse and remission cycle^19^.

Commensal microbes affect CNS inflammation both positively and negatively. Microbial dysbiosis was observed in MS patients^20–24^, and fecal transfer from MS patients to germ-free (GF) mice increased experimental neuroinflammation^25,26^. The microbiota plays complex roles in autoimmune CNS inflammation. GF and antibiotics-treated mice develop relatively mild experimental autoimmune encephalitis (EAE) compared to mice with normal microbiota^27^. In contrast, depletion of microbiota in Albino Oxford rats led to an exacerbated EAE response^28^. Suppressive effects of bacterial metabolites on inflammatory responses have been reported^29,30^. It has been reported that SCFAs have overall protective effects on EAE development^31,32^. However, it has not been determined if MS patients are deficient in SCFAs. Moreover, we hardly understand the detailed functions of SCFAs and their receptors in regulating autoimmune CNS inflammation.

We determined SCFA levels in the blood plasma from active SPMS patients and studied the roles of SCFAs, prebiotics (i.e. DF), and SCFA receptors (GPR41 and GPR43) in regulating immune cells involved in EAE pathogenesis. We found that the levels of major SCFAs are significantly decreased in the blood of long-term active SPMS patients, and SCFAs can promote IL-10-mediated anti-inflammatory activity of antigen presenting cells (APCs) and T cells. Furthermore, we found that SCFAs can also exert inflammatory effects on the CNS through effector T cells and cell surface GPCRs.

## Results

### Blood SCFA deficiency in active progressive multiple sclerosis patients

**Figure 1:**
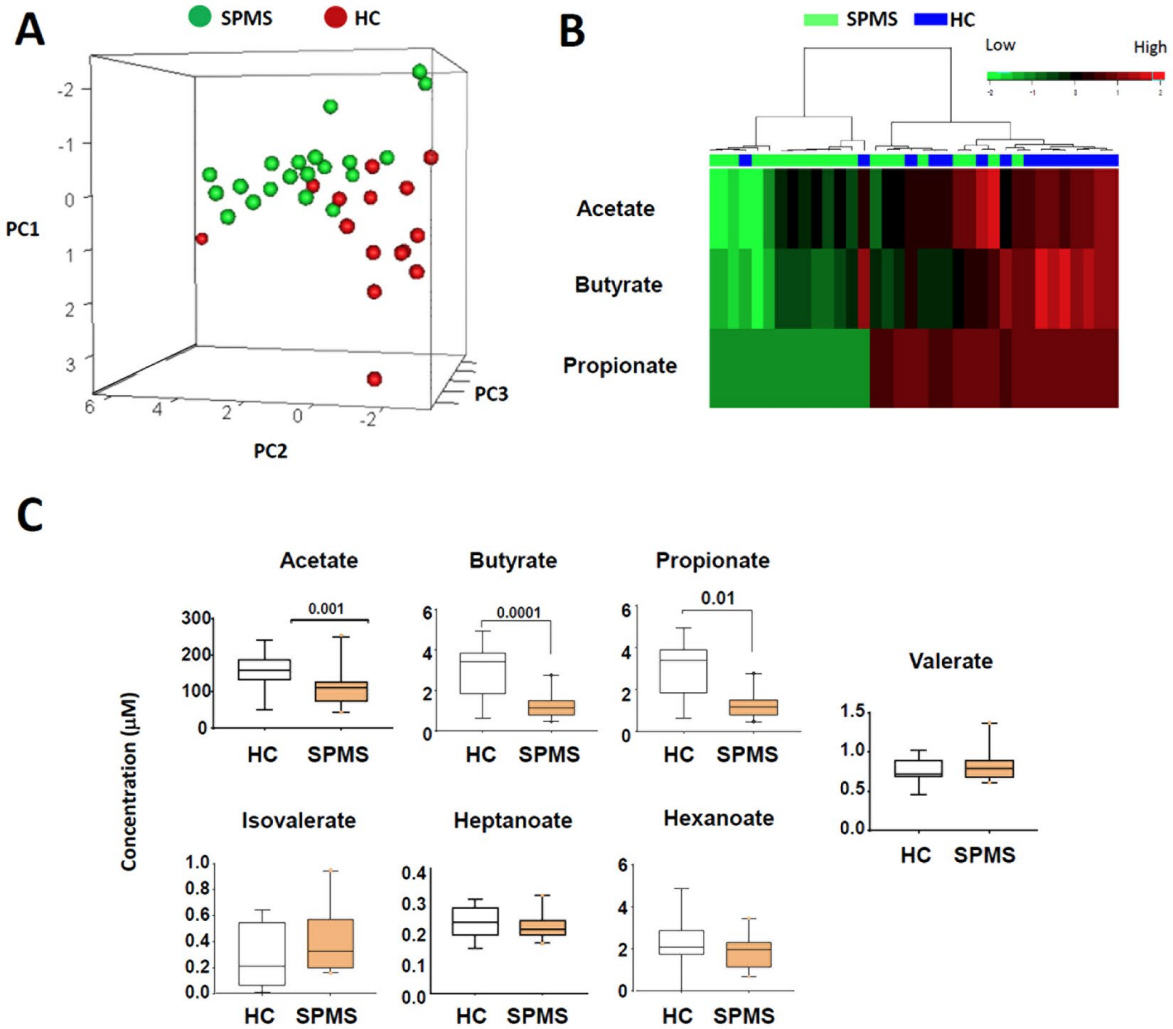
Blood levels of SCFAs in long-term MS (SPMS) patients versus healthy subjects. (**A**). Principle component analysis of the levels of 7 SCFAs determined by a targeted GC-MS approach. (**B**) A heatmap with Pearson correlation analysis for three major SCFAs. (**C**) Levels of SCFAs in the blood of SPMS patients and healthy control subjects. The data were obtained from the blood plasma of 15 healthy controls and 20 SPMS patients with comparable age ranges and male/female ratios. The boxplots display the 95% confidence intervals by Mann-Whitney test. P values are shown on top of each graph.

Despite the publication of several metabolomics studies on MS patients^33–35^, the SCFA status in autoimmune neuroinflammation has not been established in detail. Because patients with CIS or RRMS have fluctuating disease activity, we chose a group of active SPMS patients with relatively more stable disease activity for the study. These patients had active SPMS with long disease duration (15–20 years) and Expanded Disability Status Scale (EDSS) range of 3–6.5. Only the patients not treated for at least 3 months prior to the blood draw were included. The control and SPMS groups were matched for similar sex ratio and age range (average age of 52.8 ± 24.6, 25–60 years old versus average age of 52.8 ± 6.6, 35–61 years old SPMS patients). We performed a targeted GC-MS approach to measure the levels of seven different SCFAs. Compared to the healthy control group, the SPMS group has an overall distinct profile of SCFAs compared to the healthy control group (Fig. 1A). The level of propionate (C3) was decreased in ~65% of SPMS patients, compared to the control group (Fig. 1B). The level of butyrate (C4) was also decreased in 17 out of 20 patients. The level of C2 was decreased in ~50% SPMS patients. Only one out of fifteen healthy control subjects had decreased levels of C2, C3 and C4, whereas nine out of twenty SPMS had decreased levels of the three SCFA metabolites. The average levels of C2, C3, and C4 in the SPMS were 50–65% lower than those of the control group (Fig. 1C). However, no significant differences were found between the two groups for other SCFAs such as isolvalerate, valerate, hexanoate, and heptanoate.

### SCFAs increase IL-10^+^ T cells during EAE pathogenesis

**Figure 2:**
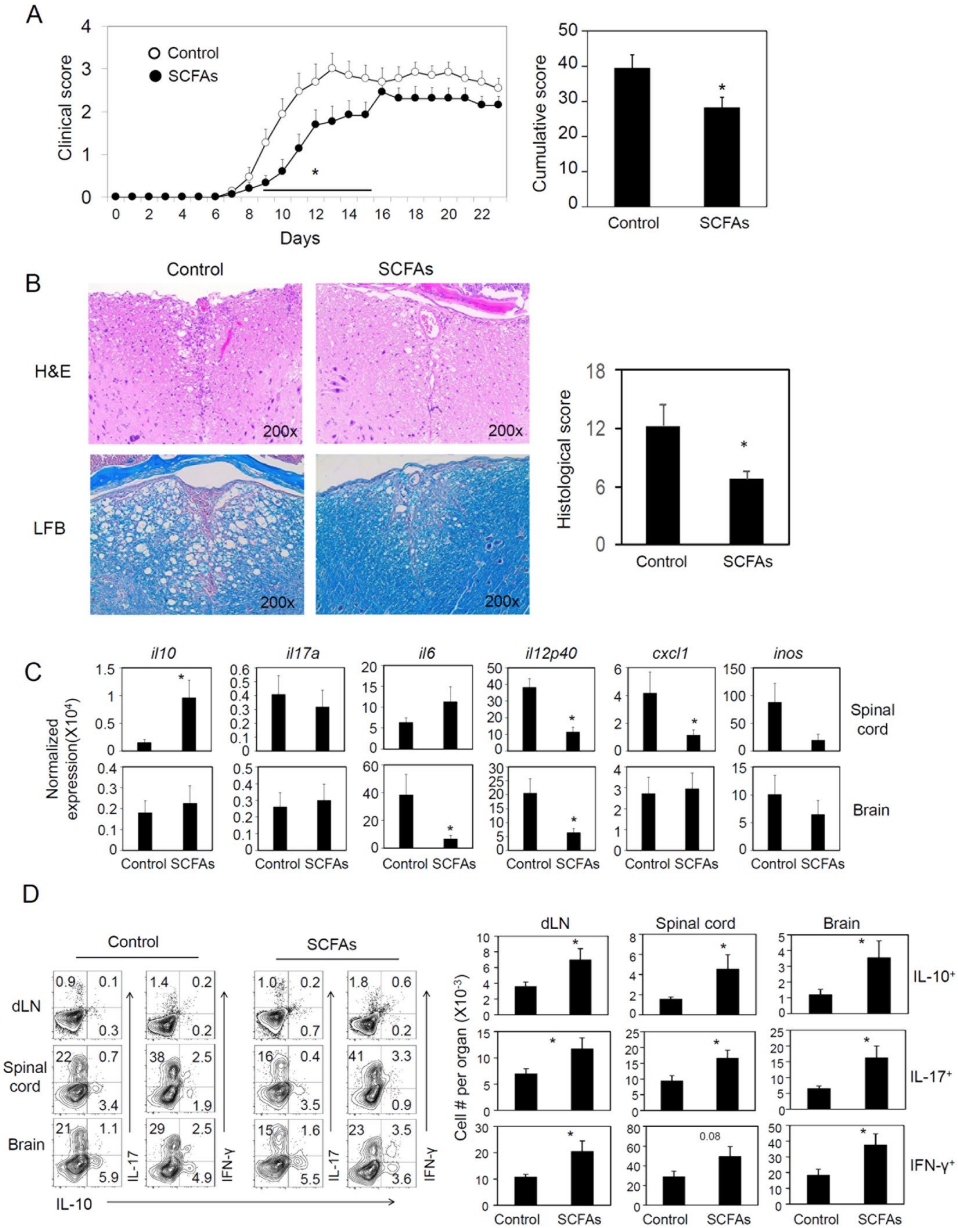
Impact of oral administration of SCFAs on EAE pathogenesis and related immune responses. (**A**) EAE clinical score of mice fed a SCFA mixture (C2 at 80 mM, C3 at 40 mM, and C4 at 20 mM) in drinking water starting from 2 weeks before MOG_35–55_ immunization and until the terminal of experiments. (**B**) Histological examination of the spinal cord. Hematoxylin and eosin (H & E) staining and luxol fast blue (LFB) myelin staining were performed. Average histological scores are shown (n = 4). (**C**) mRNA expression of cytokines and chemokines in the CNS tissues. (**D**) Representative flow dot plots and numbers of CD4^+^ T cells expressing IL-17, IFN-γ and/or IL-10^+^ cells in draining LN, spinal cord, and brain tissues. The data in panel (B–D) were obtained at the peak of EAE activity (day 11–13). Representative and pooled data obtained (mean ± SEM, n = 6–15) from 3 independent experiments are shown. *Significant differences between indicated pairs (P < 0.05).

The EAE induced by immunization with myelin oligodendrocyte glycoprotein (MOG) is a widely used animal model of MS. It has been reported that oral feeding of mice with SCFAs decreased EAE pathogenesis^31,32^. We fed mice with a mixture of SCFAs (C2 at 80 mM, C3 at 40 mM, and C4 at 20 mM), which would double the available amounts of SCFAs in the colon. As shown in Fig. 2, EAE pathogenesis, based on paralysis score (Fig. 2A) and inflammation-related spinal cord damage at the peak of EAE pathogenesis (Fig. 2B), was milder with SCFA administration. The expression of inflammatory cytokine genes such as *Il12p40, Cxcl1*, and *Inos* at the mRNA level was suppressed by SCFAs in the spinal cord. In contrast, *Il10* expression was increased in the spinal cord but not in the brain (Fig. 2C). In the brain, the expression of *Il6* and *Il12p40* was suppressed by the SCFA treatment. Increased expression of tissue *Il10* and numbers of IL-10^+^ T cells correlated with the suppressed inflammation by SCFAs. At the same time, the numbers of Th17 and Th1 effector cells were also increased by the SCFA administration (Fig. 2D). Thus, SCFAs have significant effects on both suppressive and inflammatory cytokines and T cells.

### While SCFAs directly and indirectly induce IL-10-expressing anti-inflammatory cells, they also generate inflammatory T cells

**Figure 3:**
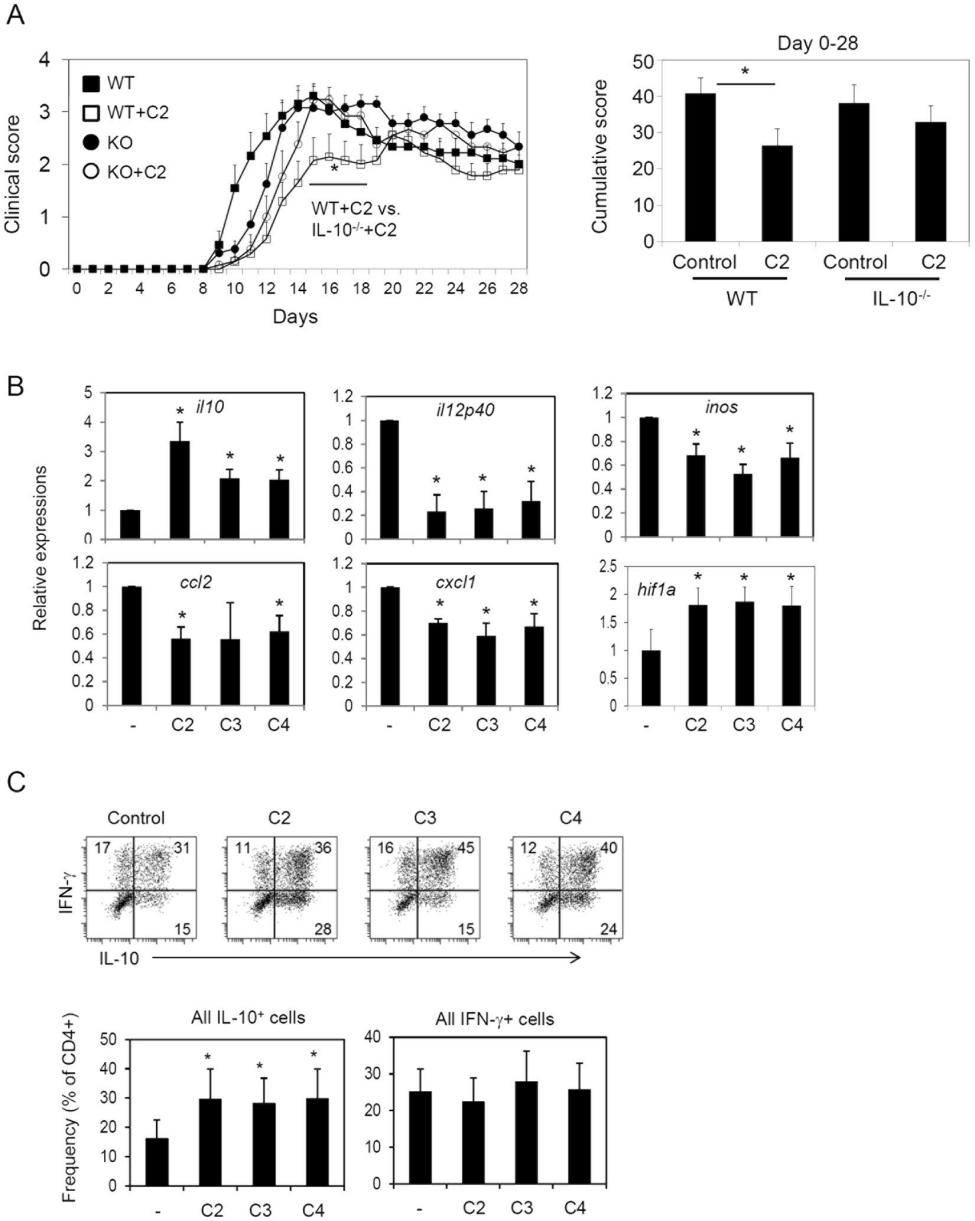
Role of IL-10 in SCFA-mediated suppression of EAE, and induction of tolerogenic antigen presenting cells by SCFAs. (**A**) C2 effect on EAE development in WT versus IL-10-deficient mice. Wild type and IL-10-deficient mice were on C2 drinking water during the whole experimental period. Immunization with MOG_35–55_ peptide was performed 2–4 weeks after the mice were on C2 water. Representative and pooled data obtained (mean ± SEM, n = 4–15) from 3–4 independent experiments are shown. *Significant differences between indicated pairs (P < 0.05). (**B**) mRNA expression of indicated genes by CNS-tissue derived glial cells cultured in the presence and absence of SCFAs (C2 at 10, C3 at 1, and C4 at 0.5 mM). The glial cells were established for 4 days *in vitro* and activated with TNF-α (10 ng/ml) and IL-17 (25 ng/ml) for 3 days. (**C**) IL-10 and IFN-γ expression by CD4^+^ T cells that were co-cultured in the presence and absence SCFA-treated glial cells. The glial cells that were activated as in panel B were co-cultured with naïve CD4^+^ T cells in the presence of SEB for 5–6 days, and the frequency of IL-10^+^ and IFN-γ^+^ T cell were examined. Representative and pooled data (mean ± SEM, n = 4–5) are shown. *Significant differences between indicated pairs (P < 0.05).

IL-10 has the potential to mediate the anti-inflammatory effect of SCFAs and has regulatory effects on EAE^36,37^. In this study, the numbers of IL-10^+^ T cells along with the expression of *Il10* in CNS tissues were increased by *in vivo* administration of SCFAs (Fig. 2). Up-regulation of IL-10 expression, but not the increased numbers of IL-17 and IFN-γ, correlated well with the protective effect of SCFAs. We examined the role of IL-10 in mediating EAE pathogenesis, utilizing IL-10^−/−^ mice fed with SCFAs in drinking water. We found that the C2-mediated suppression of EAE was modestly blunted in IL-10^−/−^ mice (Fig. 3A). These data indicate that IL-10 may mediate, in part, the protective effect of SCFAs.

SCFAs are absorbed in the colon and detected in the blood. Therefore, they can reach any organs including the CNS. We, next, investigated if SCFAs can significantly alter the immunological phenotype of APCs in the CNS. As APCs, glial cells, mainly composed of microglial cells and small numbers of non-microglial leukocytes isolated from newborn mouse brain tissues, were studied^38^. We found that all major SCFAs, such as C2, C3 and C4, have significant impacts on inflammatory cytokine (TNF-α and IL-17)-induced responses in the glial cells. The expression of *Il10* and *Hif1a* genes was increased, whereas that of inflammatory mediator genes such as *Il12p40*, *Inos*, *Ccl2*, and *Cxcl1* was suppressed by SCFAs (Fig. 3B). Because these cells can present antigens to T cells^39^, we co-cultured the SCFA-treated APCs with naive CD4^+^ T cells in the presence of SEB (a superantigen for T cell activation) and examined IL-10 expression by the T cells. We found that SCFA-treated glial cells were effective in inducing IL-10 expression in T cells (Fig. 3C). These results indicate that the protective effect of SCFAs can be mediated, in part, through APCs.

**Figure 4:**
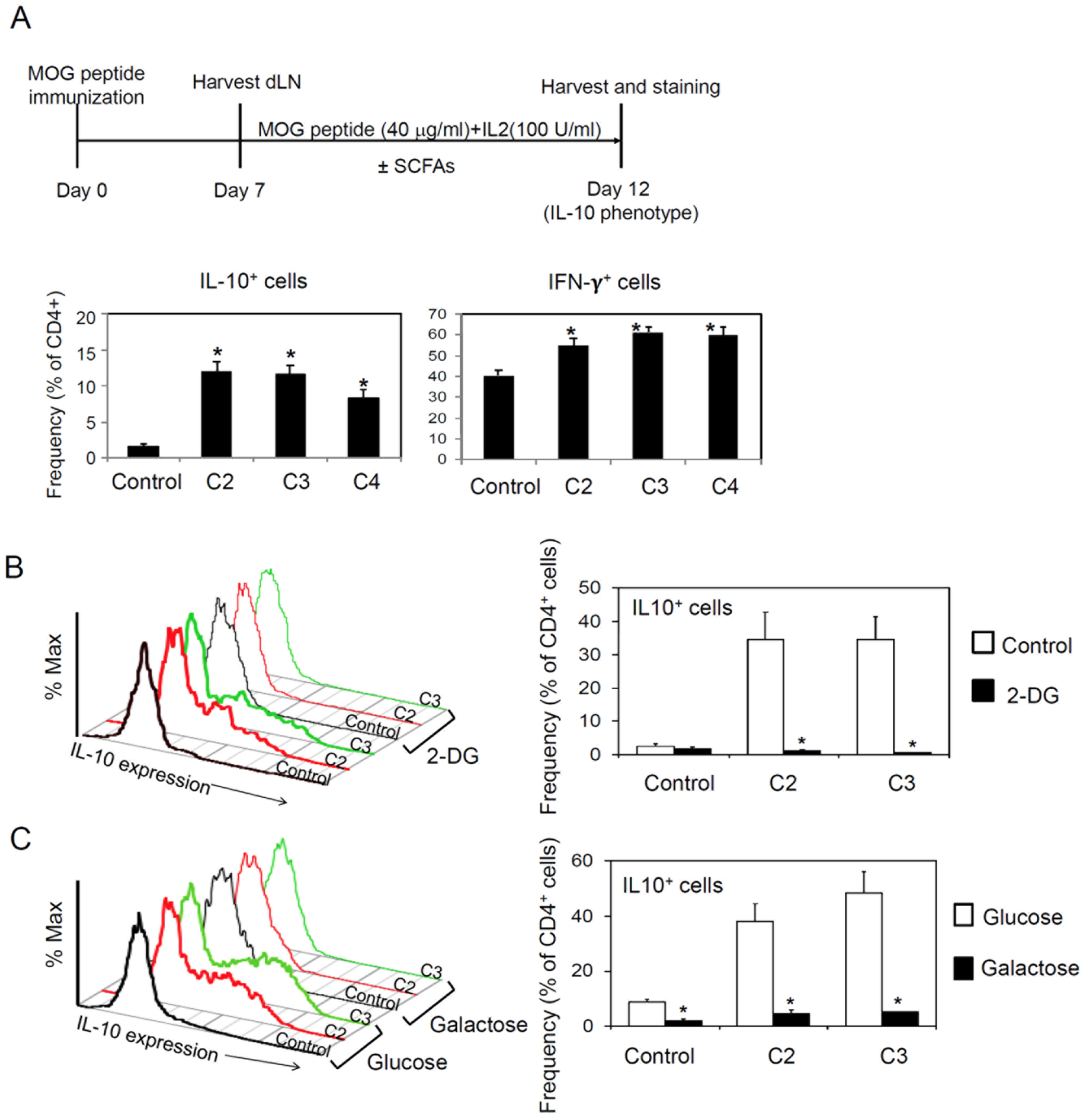
Impact of SCFAs on IL-10 expression by CD4^+^ T cells. (**A**) IL-10 and IFN-γ expression by CD4^+^ T cells, which were isolated from MOG-immunized mice and cultured in the presence or absence of SCFAs. (**B**) Impact of glycolysis inhibitor (2-DG) on SCFA-induced IL-10 expression. (**C**) Impact of galactose as the carbon source on SCFA-induced IL-10 expression. For panel A, draining lymph node cells from MOG-immunized mice were cultured in the presence of SCFAs and the MOG_35–55_ peptide for 5 days. For panel B and C, CD4^+^ T cells were activated with anti-CD3/28 with IL-2 for 5–6 days in the presence of 2-DG (0.5 mM). In panel C, glucose (10 mM) or galactose (10 mM) was used as the carbon source. Frequency of IL-10^+^ CD4^+^ T cells was determined by flow cytometry.

SCFAs can increase the expression of IL-10^40–42^. We examined if SCFAs increase IL-10 expression by CD4^+^ T cells *in vitro*. For this, we cultured the draining lymph node cells from MOG-immunized mice in the presence of SCFAs and examined expression of IL-10 and IFN-γ by CD4^+^ T cells. SCFAs increased IL-10 and IFN-γ expression by the CD4^+^ T cells *in vitro* (Fig. 4A). Because SCFAs affect lymphocyte metabolism^40,43^, we examined if glycolysis and/or mitochondrial oxidative phosphorylation are required for the boosting effect of SCFAs in IL-10 expression. We found that inhibition of glycolysis by 2-DG suppressed the IL-10 expression, and SCFAs failed to increase IL-10 expression in this condition (Fig. 4B). Moreover, T cells were not able to express IL-10 on galactose as the sole carbon source for metabolism (Fig. 4C). This indicates that mitochondrial oxidative phosphorylation without glycolysis is not sufficient for IL-10 expression, and SCFAs fail to increase IL-10 expression in this condition. Overall, the boosting effect of SCFAs on glycolysis is important for IL-10 expression by T cells.

**Figure 5:**
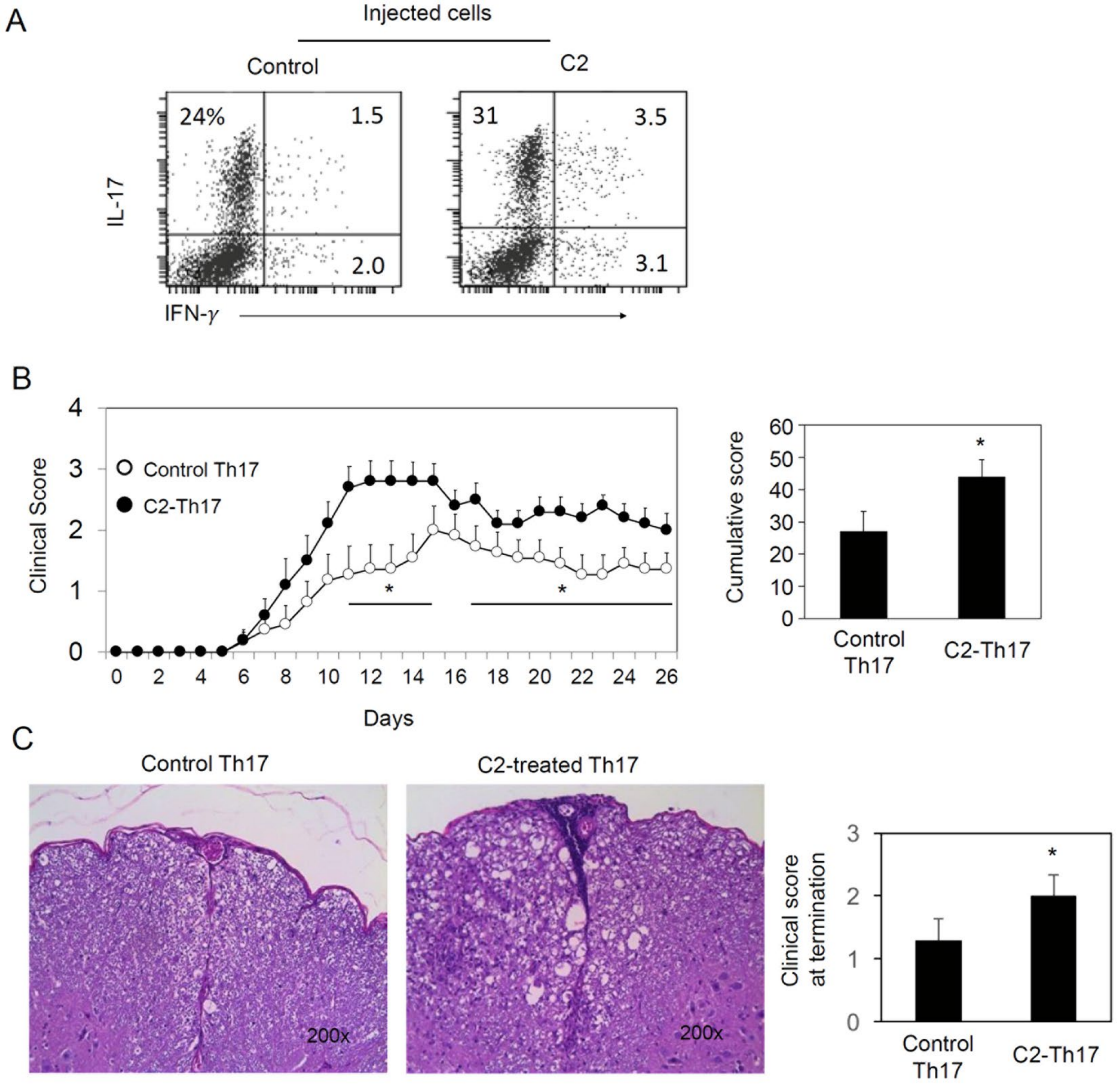
SCFA-treated T cells are inflammatory. (**A**) IL-17 and IFN-γ expression by CD4^+^ T cells, isolated from MOG-immunized mice and cultured with IL-6 and TGF-β1 in the presence and absence of C2 for 5–6 days. (**B**) Clinical score of mice injected with the SCFA-treated CD4^+^ T cells cultured in the presence or absence of C2. (**C**) Histological examination of spinal cords. Mice were immunized with MOG_35–55_ peptide. Seven days later, draining lymph node CD4^+^ T cells were cultured with MOG_35–55_ peptides (10 μg/ml) and irradiated splenocytes. hTGF-β1 (5 ng/ml, BioLegend) and mIL-6 (20 ng/ml, BioLegend) along with C2 (10 mM) were added to culture. After 5–6 days, CD45.2^+^ CD4^+^ T cells were isolated with magnetic beads (Miltenyi Biotec) and 5 × 10^6^ cells were injected i.v. into CD45.1^+^ congenic mice. Representative and pooled data obtained (mean ± SEM, n = 10–11) from 2 independent experiments are shown. *Significant differences between indicated pairs (P < 0.05).

SCFAs can also boost the generation of effector T cells such as Th1, Th17 and Tc cells depending on cytokine condition^40^. Therefore, we tested the possibility if these effector T cells generated in the presence of C2 would be inflammatory. We isolated lymph node CD4^+^ T cells from MOG-immunized mice and then reactivated them with the MOG peptide *in vitro* in the presence or absence of C2 in a Th17 cell-cytokine condition for 5–6 days. These T cells were transferred into recipient mice to induce an EAE response (Fig. 5A). The C2-treated T cells were more inflammatory than the control T cells prepared without C2 (Fig. 5B,C). Thus, the results indicate that SCFAs can also directly promote the generation of inflammatory T cells.

### The SCFA receptors GPR41 and GPR43 have pro-inflammatory roles

**Figure 6:**
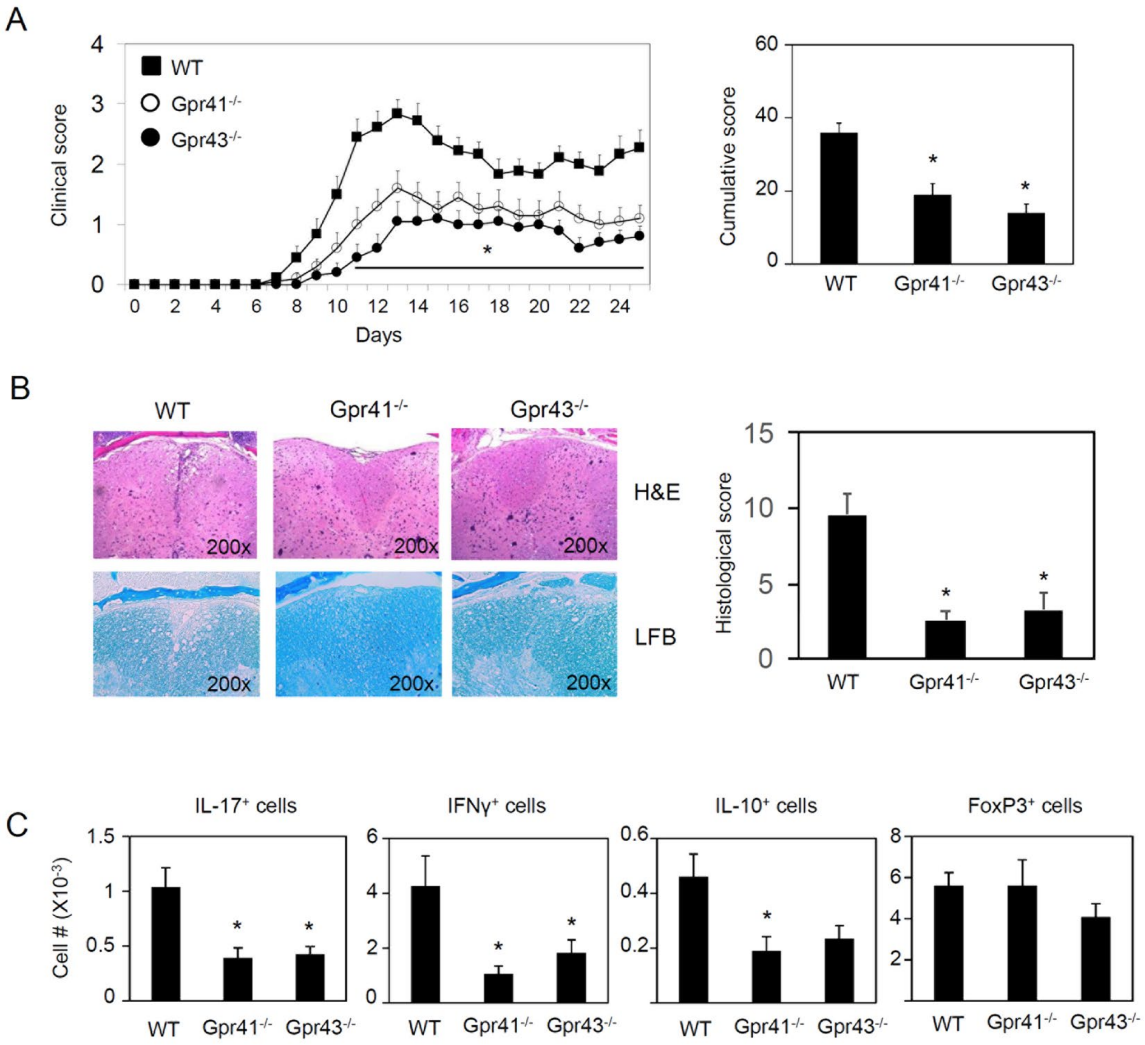
Decreased EAE responses in mice deficient in GPR41 or GPR43. (**A**) Cumulative clinical EAE score. (**B**) Histological examination of spinal cord (n = 8). (**C**) Effector and regulatory CD4^+^ T cells in the spinal cord. Representative and pooled data obtained (mean ± SEM, n = 6–15) from 2–3 independent experiments are shown. *Significant differences between indicated pairs (P < 0.05).

A part of the regulatory effect of SCFAs is mediated by GPCRs. The activation signal through GPR43 or GPR41 can mount both inflammatory and anti-inflammatory responses depending on the context and cell types^11,44–46^. We examined the EAE response of mice that are deficient in GPR41 or GPR43. Based on clinical and histological scores and infiltration by T cells (Fig. 6A–C), both GPR41^−/−^ and GPR43^−/−^ mice were relatively more resistant to EAE pathogenesis compared to WT mice. Thus, the overall proinflammatory effect mediated by SCFA receptors on EAE pathogenesis is quite different from the overall anti-inflammatory effect of SCFAs.

### DF at 5–15% levels do not significantly alter EAE pathogenesis

**Figure 7:**
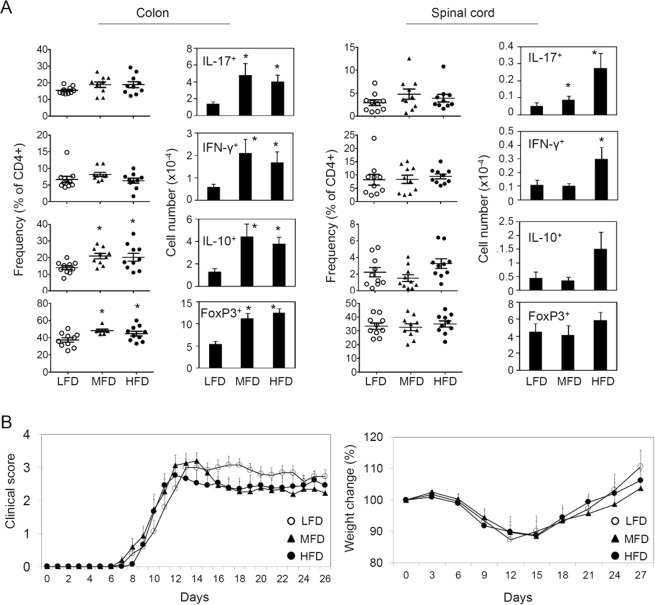
Impact of diet containing 0, 5 and 15% of combined DF (pectin and inulin = 1:1) on EAE-related T helper responses. (**A**) Frequency and number of effector and regulatory T cells were determined in indicated organs. The mice were fed indicated diet for 2 weeks before MOG immunization and until the termination of the experiments. (**B**) EAE clinical score and weight change. The data in panel A were obtained at the termination of the experiments. All diets have 5% cellulose. Representative and pooled data obtained (mean ± SEM, n = 10–15) from 3 independent experiments are shown. *Significant differences between indicated pairs (P < 0.05).

SCFAs are readily produced from soluble DF, such as pectin, inulin and arabinoxylan^37^. A previous study demonstrated that pectin at 30% of diet had a protective effect on EAE response^32^. While this is informative and in line with the protective effect of SCFAs, there is a caveat that the diets used in the study did not have insoluble DF such as cellulose that can regulate SCFA-independent processes (e.g. regulating stool bulk, transit time and others), and that 30% pectin in the high DF diet is a difficult level to naturally achieve for a prolonged time period. Moreover, the control and 30% pectin diets used in Mizuno *et al*. are quite different in calorie amounts because starch replaces pectin in the control diet, and caloric differences can also affect EAE pathogenesis as well^47^. We used diets containing more readily achievable levels of soluble DF (equal amounts of pectin and inulin for combined 15% for HFD, 5% for MFD, and 0% LFD), which creates dose-dependent differences in SCFA levels in the gut lumen and blood demonstrated in our previous studies^43^. Moreover, all of these diets contain cellulose at 5% to rule out the effect of insoluble DF deficiency. Both medium level fiber diet (MFD, 5% pectin and inulin and 5% cellulose) and high level of fiber diet (HFD, 15% pectin and inulin with 5% cellulose) significantly increased the numbers of Th17, Th1, IL-10^+^ T cells, and FoxP3^+^ T cells in the colon, compared to LFD (0% pectin and inulin and 5% cellulose, Fig. 7A). HFD, but not MFD, also increased the numbers of Th17 and Th1 cells in the spinal cord (Fig. 7A). However, we found, overall, no significant effect of the moderately different levels (0, 5, and 15%) of soluble the DF on EAE pathogenesis (Fig. 7B).

## Discussion

A mounting body of evidence indicates that gut microbiota and dietary metabolites exert their regulatory effects on CNS inflammation^48,49^. We found that the levels of three major SCFAs (C2, C3, and C4) were considerably decreased in the blood of active SPMS patients. We also systematically studied the effects of SCFAs, soluble DF, and GPCRs on EAE development in mice. We found that oral SCFA administration had a moderate protective effect on EAE pathogenesis, and this is, in part, mediated by IL-10 production in T cells and APCs induced by SCFAs. Both SCFAs and soluble DF significantly affect T cell effector phenotype. Strikingly, mice deficient in GPR43 or GPR41 were more resistant to EAE pathogenesis compared to WT mice, suggesting these SCFA receptors can promote inflammation. The results highlight multifaceted functions of the DF-SCFA-GPCR system in regulating autoimmune CNS inflammation. DF, SCFAs and their receptors appear to play highly complex functions in regulating autoimmune CNS inflammation.

Our results identify significant SCFA deficiencies in long-term active progressive MS patients. Microbial dysbiosis along with decreased numbers of C4 producers in the colon of MS patients have been reported^22,23^. In this study, the levels of all major SCFAs, such as C2, C3 and C4, were decreased in the majority of active SPMS patients. It was striking that active SPMS patients had C3 and C4 at only ~1/3 of the levels of the healthy control group. Together with the overall protective effect of SCFAs, the SCFA deficiency in patients has the potential to exacerbate the inflammation. At present, it is not clear why the SPMS patients have SCFA deficiency. One potential reason is dysbiosis or decreased levels of SCFA-producing bacteria. Changes in lifestyle and diet could lead to consumption of less DF. More studies should be performed in this regard.

We demonstrated that SCFAs promote the generation of IL-10^+^ T cells *in vitro* and *in vivo* in an EAE-inducing condition. SCFAs boost cellular metabolism by regulating HDACs and mTOR activity to induce IL-10 expression in T cells^40–42,50,51^. We also found that SCFAs promote the expression of IL-10 by conditioning CNS-resident APCs. In fact, SCFAs not only directly increased IL-10 expression by T cells but also promoted the activity of APCs to induce IL-10-producing T cells. In this regard, the protective effect of SCFAs was decreased in IL-10-deficient mice. Therefore, IL-10 appears to be a key pathway for SCFA-induced regulation of EAE pathogenesis^42^.

A caveat with the use of SCFAs in regulating CNS inflammation is that SCFAs can also increase the generation of Th17 and Th1 cells^40^. The numbers of the effector T cells were increased in animals fed on SCFA water or HFD. These effector T cells may control microbes in the gut but mediate CNS inflammation. This raises the possibility that the generation of EAE-inducing inflammatory T cells during active inflammatory responses may be even promoted by SCFAs. This possibility was indeed demonstrated in this study by the inflammatory phenotype of C2-treated T cells, which were conditioned with C2 *in vitro* but isolated from MOG-immunized mice. We consistently observed that both effector cells and IL-10^+^ T cells are induced by SCFA or DF intake. This balanced effector to regulatory T cell profile appears to be the main reason behind the overall protective effect of SCFAs. As discussed, SCFAs have the potential to promote their protective effects indirectly through APCs. For example, glial cells, such as microglial cells, have the potential to mediate the protective effect of SCFAs. We demonstrated that SCFAs generate tolerogenic glial cells which, themselves, express IL-10 and can help generate IL-10^+^ T cells. This regulatory function of gut-derived SCFAs in the CNS is somewhat reminiscent of the regulatory effect of tryptophan metabolites on microglial cells^29^.

SCFAs can ameliorate inflammatory responses through APCs in other tissues beyond the CNS system. For examples, SCFAs can condition macrophages to decrease the production of inflammatory mediators and to increase their phagocytic activity and production of anti-microbial peptides in the intestine^52,53^. SCFA receptors, such as GPR43, GPR41 and GPR109a expressed by epithelial cells or myeloid cells, function to efficiently mount acute inflammatory responses, which can work to clear invading microbes and prevent long-term inflammatory responses^45,54,55^. In this regard, our results indicate that deficiency in GPR43 and GPR41 suppresses EAE development. It has been reported that these receptors can potentiate gut barrier epithelial immune responses^11,45^. Activation of these receptors leads to efficient acute responses to barrier-crossing microbes. In this regard, the acute response to infection by gut enteric bacteria is defective in mice deficient in GPR43 or GPR41, which is somewhat similar to the attenuated EAE response in these mice.

Imbalanced SCFA functions (i.e. HDAC inhibition, metabolic regulation, versus selective GPCR activation) in the absence of GPR41 or GPR43 have the potential to alter host inflammatory responses. GPR43, GPR41 and GPR109a have overlapping but largely different ligand selectivity^3^, and this can lead to heterogeneous functions in regulating autoimmune CNS inflammation. Moreover, their signaling is heterogenous in that GPR43 and GPR41 use Gi/o pathways and inhibit cAMP production, whereas GPR43, but not GPR41, couples through Gq^15^. As exemplified by many synthetic and natural non-SCFA agonists for GPR43 and GPR41, including the GPR41-activating ketone body, β-hydroxybutyrate, produced in starvation^56,57^, there could be additional natural ligands for these receptors beyond SCFAs. This also provides a reason why the SCFA receptors have immune-regulatory functions different from that of SCFAs.

We did not find detectable protective effects of DF on the EAE pathogenesis in this study. This was puzzling in that consumption of the diet, containing pectin and inulin, increases SCFA levels in the gut and blood^43^. 5% soluble DF (pectin and inulin) led to the production of ~15 (C2), ~3 (C3), and ~3 (C4) μmol per mouse in the cecum and of 0.16 (C2), 0.017 (C3), and 0.14 (C4) μmol per ml of blood plasma^43^. The SCFA levels were decreased by 3–9 times in the cecum and by 0.2–0.5 times in the blood of mice on 0% soluble DF. There are two possible explanations for the lack of any protective effect of the DF. First, we used readily achievable levels of soluble DF (equal amounts of pectin and inulin for combined 15% for HFD, 5% for MFD, and 0% LFD), compared to the use of the very high level of DF (30% pectin) used in the study by Mizuno *et al*.^32^. Our diets are also different in that all contain 5% cellulose. Second, a recent report indicates that different DF can be distinct in their effects on inflammatory responses due to differences in their structure or more specifically glycosidic linkages, leading to the enrichment of different bacteria and production of different SCFAs. For example, pectin (a good C2-producer) can be anti-inflammatory, whereas inulin (a good C4-producer) can be pro-inflammatory in an experimental colitis^58^. It is possible that different DF or SCFAs exert heterogeneous effects on inflammation. Thus, careful assessment of the beneficial and harmful effects of DF should be carried out before applications on human patients.

Overall, our results reveal the importance of SCFAs as regulators of CNS inflammation. The levels of certain SCFAs are significantly decreased in the circulation of long-term active MS patients. SCFAs can generate tolerogenic CNS APCs, which produce IL-10 and induce anti-inflammatory T cells, and this can mediate their protective effects and potentially balance out the increased numbers of inflammatory effector T cells. The pro-inflammatory function of GPR41 and GPR43 adds to the complexity of SCFA-mediated regulation of CNS autoimmunity. Selective control of these positive and negative functions of the SCFA system presents potentially important points of intervention in autoimmune CNS diseases.

## Methods

### Patient characteristics and preparation of plasma samples

All subjects, examined for this study in the Multiple Sclerosis Center at the University of Michigan Health System, had a clinical diagnosis of secondary progressive multiple sclerosis (SPMS) and were not treated for at least 3 months prior to the blood draw. In addition, blood samples were also taken from 15 healthy controls (average age: 52.8 ± 24.6, range: 25–60 years old) with average age- and sex ratio-matched 20 SPMS patients (average age: 52.8 ± 6.6, 35–61 years old). All subjects were from Michigan and on regular diet. Blood samples were collected in tubes containing sodium citrate (BD Biosciences), and plasma samples were stored at −80 °C. Samples were thawed only once immediately prior to the analysis.

To avoid systematic bias in the analysis, all samples were randomized prior to the metabolite extraction procedure and analyzed blindly. 100 μL of plasma was transferred to a glass tube (15 × 75 mm) and 200 μL of 30 mM hydrochloric acid plus isotopically-labeled acetate (150 μM), butyrate (10 μM), and hexanoate (2 μM) in water was added to each sample, and then vortexed for 10 seconds. 300 μL of methyl tert-butyl ether (MTBE) was added to each sample, vortexed for 10 seconds to emulsify, then held at 4 °C for 5 mins, and vortexed again for 10 seconds. Samples were centrifuged for 1 minute to separate the solvent layers and the MTBE layer was then removed to an autosampler vial for GC-MS analysis. Quality control (QC) samples were prepared by pooling equal volumes of each sample and injected at the beginning and the end of each analysis and after every 10 sample injections to provide a measurement of the system’s stability and performance as well as reproducibility of the sample preparation method. A series of calibration standards were prepared along with samples to quantify metabolites.

### Measurements of metabolites

Gas chromatography–mass spectrometry (GC-MS) analysis was performed on an Agilent 69890 N GC-5973 MS detector with the following parameters: 1 µL of samples was injected with a 1:10 split ratio on a ZB-WAXplus GC column (30 m × 0.25 mm × 0.25um, Cat#7HG-G013-11, Phenomenex), with He as the carrier gas at a flow rate of 1.1 ml/min. The injector temperature was 240 °C, and the column temperature was isocratic at 310 °C. Data were processed using MassHunter Quantitative analysis (version B.07.00). SCFAs were normalized to the nearest isotope-labeled internal standards and quantitated using 2 replicated injections of 5 standards to create a linear calibration curve with accuracy better than 80% for each standard. SCFAs were normalized to the nearest isotope labeled internal standard and quantitated using 2 replicated injections of 5 standards to create a linear calibration curve with accuracy better than 80% for each standard.

### Mice, diet and SCFA administration

IL-10^−/−^ mice on the C57BL/6 background were from the Jackson Laboratory. Mice deficient in GPR43 or GPR41 on the C57BL/6 background were previously described^11^. Mice were fed regular rodent chow (Harlan 2018S Global 18% Protein Rodent Diet) or special DF diets from the Envigo (Indianapolis, IN). The special diet contained 5% of cellulose but they had different amounts of soluble DF (0% of pectin and inulin at 1:1 ratio for LFD, 5% for MFD, and 15% for HFD). WT and IL-10^−/−^ mice were fed the special diet from 3 weeks of age and/or drinking water containing sodium acetate (C2 at 200 mM, pH 7.4) or a SCFA mixture (C2 at 80 mM, C3 at 40 mM, and C4 at 20 mM, pH 7.4, Sigma-Aldrich) for 2–4 weeks before induction of EAE until the termination of the experiments.

### Experimental autoimmune encephalomyelitis (EAE) in mice

For induction of EAE, 8–10-week-old female mice were immunized with the MOG_35–55_ peptide (100 μg/mouse, AnaSpec) in complete Freund’s adjuvant (Difco Laboratories). On day 0 and day 2, pertussis toxin (100 ng/mouse, List Biological Labs) was injected intraperitoneally. EAE was also induced with adoptive transfer of MOG-stimulated T cells. For this, C57BL/6 mice were immunized with MOG_35–55_ peptide. Seven days later, total CD4^+^ T cells were isolated from draining LNs, and cultured with MOG_35–55_ peptides (10 μg/ml) and irradiated splenocytes. hTGFβ1 (5 ng/ml, BioLegend) and mIL-6 (20 ng/ml, BioLegend) along with C2 (10 mM) were added to culture. After 5–6 days, CD4^+^ T cells were isolated with magnetic beads (Miltenyi Biotec) and 5 × 10^6^ cells were injected i.v. into CD45.1^+^ congenic C57BL/6 mice. Spinal cords were embedded in paraffin, cut into 6 μm sections, and stained with hematoxylin (HnE) and eosin or luxol fast blue (LFB). Spinal cords were embedded in paraffin, cut into 6 μm sections, and stained with HnE or LFB (0.1%, Sigma-Aldrich). For LFB staining, paraffin sections were stained with a LFB solution (0.1%) overnight at 56 °C and the slides were differentiated in lithium carbonate solution (0.05%) for 30 seconds and counterstained with cresyl violet solution (0.1%) for 30 seconds. EAE activity was scored as following: no overt signs of disease (0); limp tail or hind limb weakness but not both (1); limp tail and hind limb weakness (2); partial hind limb paralysis (3); complete hind limb paralysis (4); moribund state; and death by EAE (5). Histological scores were obtained based on hematoxylin and eosin staining of formalin-fixed spinal cords. Three spinal cord sections at high, middle and low (cervical, thoracic and lumbar) positions from each mouse were examined and the tissue section with the highest score was chosen for each mouse for scoring. Scoring (1–5) was based on the numbers and size of lesions defined by both immune cell infiltration and tissue structure destruction/disorganization.

### Flow cytometry

Spinal cord and brain tissues were harvested and digested with collagenase type 4 (2 mg/ml, Worthington Biochemical) for 1 hour at 37 °C in RPMI medium supplemented with 10% FBS. For T cell characterization by flow cytometry, cells were stained with antibodies to CD4 (clone RM4-5, BioLegend). Intracellular staining of the cells was performed after activation with phorbol myristate acetate, ionomycin and monensin and stained with antibodies to IL-10 (clone JES5-16E3, BioLegend), IL-17A (clone TC11-18H10.1, BioLegend), or IFN-γ (clone XMG1.2, BioLegend).

### Cell isolation and culture

Naïve CD4^+^ T cells were isolated from splenocytes and lymph node cells with the naive CD4^+^ T cell isolation kit (Miltenyi Biotec). To obtain CNS APCs, brains from newborn mice were collected, dissociated with scissors and digested with collagenase type 4 (2 mg/ml, Worthington Biochemical) for 1 hour at 37 °C. Mononuclear cells in the interphase between 30% and 70% of Percoll (GE healthcare) were isolated by density-cut centrifugation as described^38^. These cells were cultured for 4 days and then activated by TNF-α (10 ng/ml, BioLegend) and IL-17 (25 ng/ml, BioLegend) in the presence and absence of SCFAs (C2 at 10 mM, C3 at 1 mM, C4 at 0.5 mM) for additional 3 days in DMEM medium (10% FBS). Adherent glial cells, largely composed of microglial cells, were co-cultured as APCs with naïve CD4^+^ T cells at 1:10 ratio for 5–6 days in the presence of Staphylococcal enterotoxin B (SEB, 5 μg/ml, List Labs) to stimulate naïve CD4^+^ T cells isolated from the spleen and lymph nodes of unimmunized C57BL/6 mice. For flow cytometry of cytokine expression by T cells, total draining LN cells from MOG-immunized mice were cultured with MOG_35–55_ peptide (40 μg/ml) and hIL-2 (100 U/ml) in the presence or absence of SCFAs (C2, 10 mM; C3, 1 mM; C4, 0.5 mM) for 5 days.

### Metabolic impact of SCFAs on T cells

Naïve CD4^+^ T cells were activated with plate-coated anti-CD3 (5 μg/ml) and soluble anti-CD28 (2 μg/ml) for 5–6 days in the presence of C2 (10 mM) or C3 (1 mM). Regular RPMI 1640 medium (10% FBS) and glucose-free RPMI 1640 medium (10% dialyzed FBS) supplemented with glucose (10 mM) or galactose (10 mM) were used. 2-Deoxy-D-glucose (2-DG, 0.5 mM) was added as an inhibitor of glycolysis when indicated.

### Quantitative real-time PCR

Using TRIzol® solution (Invitrogen), total RNA was extracted, and cDNA was synthesized with SuperScript® II Reverse Transcriptase (Invitrogen). Quantitative real-time PCR (qRT-PCR) was performed with Maxima® SYBR Green/ROX qPCR Master Mix (Thermo Scientific). The primers used for the qRT-PCR were: CTG.GTC.TTC.TGG.AGT.ACC.ATA.GC and TGC.CGA.GTA.GAT.CTC.AAA.GTG.AC for *Il6*; CCA.GCT.GGA.CAA.CAT.ACT.GCT and CAT.CAT.GTA.TGC.TTC.TAT.GCA.G for *Il10*; GAC.TCT.CCA.CCG.CAA.TG and CGG.GTC.TCT.GTT.TAG.GCT for *Il17a*; GAA.CTG.GCG.TTG.GAA.GCA.CG and CTA.GGA.TCG.GAC.CCT.GCA.GG for *Il12p40*; GCG.GGT.ACC.ATG.AAG.ATC.TC and CAG.GGT.CAG.AAT.CAA.ACC.CT for *Cxcl1*; GGA.AGA.AAT.GCA.GGA.GAT.GGT and CTG.TCA.GAG.CCT.CGT.GGC.TT for *Inos*; AAG.ATG.TGC.TGG.ACA.GCT.G and TCC.AGG.GCA.CAT.ATG.CAG.AG for *Ccl2;* GGA.ACA.TGA.TGG.CTC.CCT.TTT.TC and CAC.CCT.GCA.GTA.GGT.TTC.TGC for *Hif1a*.

Expression levels of the genes were normalized by β-actin levels.

### Statistical analysis

Student’s *t* test (1 or 2-tailed) and Mann-Whitney test were used to determine the significance of differences between two groups. The paralysis score and weight change were analyzed with repeated measures for ANOVA (SAS, version 9.2, SAS Institute). *P* values < or = 0.05 were considered significant.

### Study approval

All methods were performed in accordance with the relevant institutional guidelines and regulations. All animal experiments were approved by Animal Care and Use Committee at Purdue University. All human subjects gave written informed consent in accordance with the Declaration of Helsinki. The protocol (HUM00066792) was approved by the University of Michigan Institutional Review Board.
