# Author Correction: Bidirectional regulatory potentials of short-chain fatty acids and their G-protein-coupled receptors in autoimmune neuroinflammation

**DOI:** 10.1038/s41598-019-54276-x

**Published:** 2019-11-20

**Authors:** Jeongho Park, Qin Wang, Qi Wu, Yang Mao-Draayer, Chang H. Kim

**Affiliations:** 1 Department of Comparative Pathobiology, Purdue University, West Lafayette, IN 47907 USA; 2 Autoimmunity Center of Excellence, Multiple Sclerosis Center, Department of Neurology, University of Michigan Medical School, Ann Arbor, MI 48109 USA; 3 Laboratory of Immunology and Hematopoiesis, Department of Pathology and Mary H Weiser Food Allergy Center, University of Michigan School of Medicine, Ann Arbor, MI 48109 USA

Correction to: *Scientific Reports* 10.1038/s41598-019-45311-y, published online 20 June 2019

This Article contains errors in the Results section under subheading ‘DF at 5-15% levels do not significantly alter EAE pathogenesis’.

“Moreover, all of these diets contain cellulose at 5% to rule out the effect of insoluble DF deficiency. Both medium level fiber diet (MFD, 5% pectin and inulin and 5% cellulose) and high level of fiber diet (HFD, 15% pectin and inulin with 5% cellulose) significantly increased the numbers of Th17, Th1, IL-10^+^ T cells, and FoxP3^+^ T cells in the colon, compared to LFD (0% pectin and inulin and 5% cellulose, Fig. 7A). HFD, but not MFD, also increased the numbers of Th17 and Th1 cells in the spinal cord (Fig. 7A).”

should read:

“Moreover, all of these diets contain cellulose of at least 2.5% to rule out the effect of insoluble DF deficiency. Both medium level fiber diet (MFD, 5% pectin and inulin and 5% cellulose) and high level of fiber diet (HFD, 15% pectin and inulin with 2.5% cellulose) significantly increased the numbers of Th17, Th1, IL-10^+^ T cells, and FoxP3^+^ T cells in the colon, compared to LFD (0% pectin and inulin and 10% cellulose, Fig. 7A). HFD, but not MFD, also increased the numbers of Th17 and Th1 cells in the spinal cord (Fig. 7A).”

Additionally, in this Article, the legend of Figure 7B is incorrect,

“EAE clinical score and weight change. The data in panel A were obtained at the termination of the experiments. All diets have 5% cellulose. Representative and pooled data obtained (mean ± SEM, n = 10–15) from 3 independent experiments are shown. *Significant differences between indicated pairs (P < 0.05).”

should read:

“EAE clinical score and weight change. The data in panel A were obtained at the termination of the experiments. All diets have at least 2.5% cellulose. Representative and pooled data obtained (mean ± SEM, n = 10–15) from 3 independent experiments are shown. *Significant differences between indicated pairs (P < 0.05).”

Furthermore, in the Discussion section,

“Our diets are also different in that all contain 5% cellulose.”

should read:

“Our diets are also different in that all contain at least 2.5% cellulose.”

Lastly, this article also contains errors in the Methods section under subheading ‘Mice, diet and SCFA administration’.

“The special diet contained 5% of cellulose but they had different amounts of soluble DF (0% of pectin and inulin at 1:1 ratio for LFD, 5% for MFD, and 15% for HFD).”

should read:

“The special diet contained at least 2.5% of cellulose but they had different amounts of soluble DF (0% of pectin and inulin at 1:1 ratio for LFD, 5% for MFD, and 15% for HFD).”

